# Modulation of the Aβ-Peptide-Aggregation Pathway by Active Compounds From *Platycladus orientalis* Seed Extract in Alzheimer’s Disease Models

**DOI:** 10.3389/fnagi.2020.00207

**Published:** 2020-08-14

**Authors:** Li Yan, Xiang He, Yufan Jin, Jiawei Wang, Fengying Liang, Rongrong Pei, Peibo Li, Yonggang Wang, Weiwei Su

**Affiliations:** Guangdong Key Laboratory of Plant Resources, Guangdong Engineering and Technology Research Center for Quality and Efficacy Reevaluation of Post-Market Traditional Chinese Medicine, School of Life Sciences, Sun Yat-sen University, Guangzhou, China

**Keywords:** Aβ-peptide aggregation, *Platycladus orientalis* seed, cognitive function, isocupressic acid, β-secretase 1

## Abstract

Alzheimer’s disease (AD) is a neurodegenerative disease characterized by neuronal loss, cognitive impairment, and aphasia. Aggregation of β-amyloid (Aβ) peptide in the brain is considered a key mechanism in the development of AD. In the past 20 years, many compounds have been developed to inhibit Aβ aggregation and accelerate its degradation. *Platycladus orientalis* seed is a traditional Chinese medicine used to enhance intelligence and slow aging. We previously found that *Platycladus orientalis* seed extract (EPOS) inhibited Aβ-peptide aggregation in the hippocampus and reduced cognitive deficits in 5×FAD mice. However, the mechanisms of these effects have not been characterized. To characterize the protective mechanisms of EPOS, we used a transgenic *Caenorhabditis elegans* CL4176 model to perform Bioactivity-guided identification of active compounds. Four active compounds, comprising communic acid, isocupressic acid, imbricatolic acid, and pinusolide, were identified using 13C-and 1H-NMR spectroscopy. Furthermore, we showed that isocupressic acid inhibited Aβ generation by modulating BACE1 activity *via* the GSK3β/NF-κB pathway in HEK293-APPsw cells. These findings showed that EPOS reduced cognitive deficits in an AD model *via* modulation of the Aβ peptide aggregation pathway.

## Introduction

Alzheimer’s disease (AD) is a chronic neurodegenerative disease characterized by abnormal amyloid-β (Aβ) peptide accumulation, tau hyperphosphorylation, and neuronal loss. These neuropathologies affect brain regions involved in memory and cognition (Montine et al., 2012). Several hypotheses have been proposed for the mechanisms of AD pathology, but the Aβ cascade hypothesis has received the most attention (Frautschy and Cole, 2010).

Abnormal metabolism of Aβ plays a key role in the pathogenesis of AD (LaFerla et al., 2007). An imbalance between Aβ production and clearance results in Aβ aggregation and deposition, which triggers a pathophysiological cascade of events that includes synaptic dysfunction, tau hyperphosphorylation, and neuronal loss, which results in cognitive impairment (Janus et al., 2000; Kamenetz et al., 2003; Huang and Jiang, 2009). Amyloid-β is formed by sequential cleavage events of β-amyloid precursor protein (APP) by β-secretase 1 (BACE1) and γ-secretase (Vassar, 2004; Agostinho et al., 2015). Chen et al found that levels of nuclear factor kappa-B (NF-κB) p65 and BACE1 were higher in brains of patients with AD, and also showed that BACE1 gene expression and APP metabolism were facilitated by NF-κB p65 (Chen et al., 2012). Chen et al also showed that overexpression of NF-κB p65 increased human BACE1 promoter activity (Chen et al., 2012). Another study found that the GSK3β/NF-κB signaling pathway regulated BACE1 gene transcription (Ly et al., 2013). This finding led to a contribution that AD may develop through GSK3β/NF-κB-mediated BACE1 expression.

Medicinal herbs and natural products are significant sources of compounds that protect against AD (Kim et al., 2010). For example, curcumin, huperzine A, and EGb 761 (Ginkgo Biloba extract) have been hypothesized to protect against AD through anti-oxidative, anti-amyloidogenic, and anti-inflammatory effects (Lim et al., 2001; Zhang and Tang, 2006; Bate et al., 2008). The seed of *Platycladus orientalis* is a traditional Chinese medicine that slows aging and enhances intelligence (Inoue et al., 1985). Modern studies have also shown that it can induce sedative and hypnotic effects, and ameliorate cognitive deficits caused by amygdala lesions in mice (Sun et al., 2010). The seed of *Platycladus orientalis* has also been shown to slow physiological aging and to prevent age-related memory deficits in humans. In a previous study, *Platycladus orientalis* seed extract (EPOS) significantly prolonged the lifespan of *Caenorhabditis elegans* and protected the transgenic nematode CL4176 strain against Aβ toxicity (Liu et al., 2013).

In this study, EPOS was verified that it could significantly improve spatial working memory and reduce Aβ42 plaque deposition and alter the dendritic spine density of neurons in the hippocampus. Then, we isolated and identified the major fractions of EPOS that protected against Aβ peptide aggregation using bioassay-guided fractionation. This screening resulted in the identification of four compounds that were further evaluated for anti-Aβ aggregation effects in HEK293-APPsw cells. Among these compounds, isocupressic acid showed the best anti-Aβ generation activity by modulating BACE1 activity *via* the GSK3β/NF-κB pathway in HEK293-APPsw cells.

## Materials and Methods

### Plant Material and Extraction of *Platycladus orientalis* Seed

*Platycladus orientalis* seeds were purchased from the Guangzhou Daxiang, Limited (Guangdong, China) and were authenticated by Guangdong Institute for Drug Control (GDGID No. 2018A03174). *Platycladus orientalis* seed (0.5 kg) was powdered for 5 min and extracted three times with 2.5 volumes of petroleum ether (PE) at room temperature. The combined PE extract was concentrated to dryness in a vacuum (PE extract weight: 0.14 kg), and the resulting residue was dried on an iron plate for 1 day. The residue was refluxed with 95% ethanol (v/v) for 2 h. The combined ethanol extracts were then concentrated using a rotary evaporator at 50°C to produce EPOS.

### Animals

Transgenic mice (5×FAD) that overexpress human APP and PS1 with 5 familial AD (FAD) mutations [APP K670N/M671L (Swedish) + V717I (London) + I716V (Florida) and presenilin [(PS1) M146L + L286V] (Oakley et al., 2006) were purchased from Model Animal Research Center of Nanjing University [Certification No. SCXK-(Su) 2010-0001] and housed in a specific pathogen-free environment at the Ocean and Traditional Chinese Medicine Laboratory of Sun Yat-sen University [License No. SCXK-(Yue) 2014-0020]. All animal experimental procedures in this study were approved by “The Animal Care and Use Committee of the School of Life Sciences of Sun Yat-sen University” (Permission No.150207). The resulting progeny [hybrid 5×FAD transgenic and non-transgenic wild type (WT)] were used in this study. Genotyping was performed by polymerase chain reaction (PCR) of mouse tail DNA (Oakley et al., 2006). In this study, mice were used and handled according to the guidelines of China for animal care. Animals were kept in a temperature-controlled room at 20°C ± 2°C and a 12/12 h light/dark cycle (light 0 at 6 AM). Food and water were available *ad libitum*.

### Experimental Animal Model

Twenty-four 5×FAD Tg mice were separated into the following groups: Tg, EPOS (100 mg/kg/day), and EGb761 (100 mg/kg/day). Eight WT mice were used as controls. The mice were administered EPOS by gavage once daily for 30 consecutive days. The WT group and the Tg group were treated with the same volume of normal saline.

### Behavioral Test

The Y maze is a three-part radial box, which consists of three arms of the same length. The mice were placed at the center of the three arms and allowed to explore freely for 5 min. When the limbs of the mice were completely inserted into one arm, this was considered one entry. The total number of times and the order of arm entry were recorded. Spontaneous alternations were defined as consecutive triplets of different arm choices.

The Morris water maze consists of a circular pool made of stainless steel with a diameter of 100 cm and a height of 50 cm, with a movable platform. The platform and the pool wall were white. During the experiment, the pool and the surrounding environment remained unchanged. The pool was monitored using a computer to track and record the swimming trajectory. The Morris water maze was divided into four quadrants, and the platform was placed in the middle of the first quadrant. Swimming was recorded and data were analyzed using JLBehv-MWMM. The navigation test was conducted for 5 days, and the mice were trained once per day. The mice were placed in water for 60 s in order of consecutive quadrants. The mice were placed in the water facing the pool wall. The instrument automatically recorded the mice took looking for and climbing the platform, and the time from which the mice entered the water to the time at which the found and climbed the platform (escape the incubation period). Mice that did not find the platform within 60 s were placed on the platform for 30 s, and the time to finding the platform was recorded as 60 s. After the training period was completed, the mice were returned to their cages. On the sixth day, the spatial exploration experiment was performed. The platform in the first quadrant was removed, and the mice were placed in the water in the third quadrant and allowed to swim for 60-s. The time spent in the first quadrant was recorded for 60 s.

### Immunofluorescence Staining of Frozen Sections

Three mice per group were used for immunofluorescence staining. Mice were transcranial perfused with 4% paraformaldehyde (PFA) in PBS, and the brains were carefully dissected. The brains were fixed in 4% PFA at 4°C overnight and dehydrated using 10%, 20%, and 30% sucrose solutions for 1 day each. The brains were sliced into 20-μm sections and immunostained with rabbit anti-Aβ(1–42) IgG antibody (1:500) at 4°C overnight, then incubated with Goat Anti-Rabbit IgG (Alexa Fluor^®^ 594). Images were captured using a Nikon confocal microscope.

### Golgi Staining

Mouse brains were dissected and prepared using the FD Rapid Golgi Stain Kit (FD Neuro Technologies, Shanghai, China) according to the manufacturer’s instructions. Slices were visualized using a Nikon confocal microscope (Nikon, A1R HD25). Images of Golgi-stained hippocampal CA1 pyramidal neurons were captured. We used three animals per group to do Golgi-staining. Thirty neurons from three mice (10 slices of every sample) were analyzed for dendritic spine density by NIS-Elements Viewer (Nikon). The analyzed method was followed as previous research (Du, 2019).

### Isolation and Characterization of EPOS

Sixty-five grams of EPOS was isolated using silica gel low pressure column chromatography (Teledyne ISCO RF, USA; column: Buch, φ 10 × 25 cm, USA) with chloroform: methanol (100:0, 98:2, 95:5, 90:10, 80:20; 70:30, 60:40, and 0:100). Ten fractions were obtained (Fr1, 32.49g, 50%; Fr2, 3.48g, 5.4%; Fr3, 3.12g, 4.8%; Fr4, 1.96g, 3.0%; Fr5, 4.79g, 7.4%; Fr6, 7.24g, 11.1%; Fr7, 4.42g, 6.8%; Fr8, 2.95g, 4.5%; Fr9, 3.21g, 4.9%; Fr10, 1.34g, 2.1%).

Fraction 3 (1.5 g) was fractionated using an octadecyl silane column (50 μm, Ultimate XB-C18, USA) and eluted with methanol:H_2_O (30:70, 50:50, 30:70, 0:100). Preparative HPLC (Shimadzu, SHIMADZU-20A, Japan; column: 10 × 250 mm, Xterra C18, 5 μm, Waters, USA) was used to isolate four compounds. The structures of these compounds were determined by 13C-NMR (100 MHz) spectra and 1H-NMR (400 MHz) using a Bruker Avance 400 spectrometer. Tetramethylsilane (TMS) was used as an internal standard and CDCl_3_ as the solvent.

### *Caenorhabditis elegans* Strains and Maintenance

The *C. elegans* CL4176 strain [genotype: smg1(cc546ts); dvIs27] and *Escherichia coli* OP50 strain were obtained from the Caenorhabditis Genetics Center (University of Minnesota, Minneapolis, MN, USA; Sutphin and Kaeberlein, 2009). The CL4176 nematode model expresses human Aβ1–42 in body wall muscle cells by activating the myo-3 body wall-specific myosin promoter. When Aβ is sufficiently expressed, the nematodes produce sputum until death (Ewald and Li, 2010). We maintained *C. elegans* on nematode growth medium (NGM) agar plates with *Escherichia coli* OP50 at 16°C.

### Paralysis Assay

The above strain was egged synchronized onto the culture mediums with or without different ingredients. Transgenic expression was induced by adjusting the temperature (16–26°C). Induction occurred within 36 h after spawning and was sustained until the last worm became paralyzed. For the paralysis assay, Nematodes were marked as dead when they got the worst of responding to repeated touching with a platinum wire. Each experiment was double-blinded and performed in triplicate.

### Cell Culture, ELISA Assay, and Western Blot

WT human embryonic kidney 293 cells (HEK293/WT) and HEK293 cells stably transfected with human APP Swedish mutant (HEK293sw) were gifts from Professor Zhong Li (Sun-Yat-sen University, Guangzhou, China). The cells were maintained in Dulbecco’s Modified Eagle Medium (DMEM) with 5% fetal bovine serum in a 5% CO_2_ atmosphere at 37°C.

Following 24 h drug treatment, Aβ (1–42) in the medium was measured using an ELISA kit (CUSABIO, Wuhan, China) according to the manufacturer’s protocol. Protein content was determined using a BCA Protein Assay Kit (Beyotime Biotechnology). Levels of APP, BACE1, Aβ (1–42), ADAM10, PSEN1, NF-κB p68, GSK-3β(Tyr 216), and GSK-3β were analyzed using western blot (primary antibodies: anti-APP, 1:1,000, CST; anti-BACE1, 1:1,000, CST; anti-Aβ (1–42), 1:2,000; CST; anti-ADAM10, 1:1,000, CST; anti-PSEN1, 1:1,000, CST; anti-NF-κB p68, 1:1,000, CST; anti- GSK-3β, 1:1,000, CST; anti-GSK-3β (Tyr 216, 1:1,000, Thermo Fisher). Densitometry was performed using a Tanon5200.

### Statistical Analysis

All data are presented as the mean ± standard error of the mean (SEM), and experiments were performed in triplicate. Statistical analyses were determined using one-way ANOVA, followed by Fisher’s protected least significant difference for *post hoc* comparisons. *P*-values < 0.05 were considered significant. GraphPad Prism 7 was used for all statistical analyses.

## Results

### *Platycladus orientalis* Seed Extract Ameliorated Cognitive Deficits in 5×FAD Tg Mice

**Figure 1:**
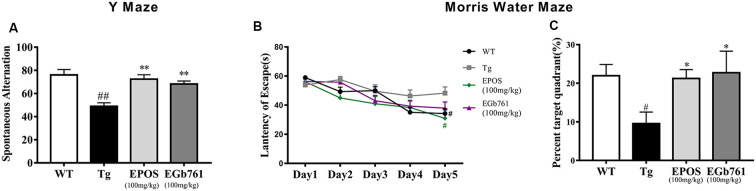
Treatment with *Platycladus orientalis seed extract* (EPOS) improved cognitive function in transgenic 5×FAD mice. **(A)** Mice [wild type (WT), Tg, Tg-EPOS, and Tg-EGb761] were tested for the spontaneous alternation in the Y maze. **(B,C)** Mice (WT, Tg, Tg-EPOS, and Tg-EGb761) were tested for the spatial learning and memory in the Morris water maze. **(B)** Escape latencies during training. ^#^*p* < 0.05 compared with Tg. **(C)** In the probe test, the time spent in the quadrant with the hidden platform was measured. ^#^*p* < 0.05 compared with WT, ^##^*p* < 0.01 compared with WT, *p < 0.05 compared with Tg, **p < 0.01 compared with Tg, compared with model. Data are presented as the mean ± SEM, *n* ≥ 8 mouse per group (one-way ANOVA).

To investigate whether EPOS ameliorated cognitive deficits in Tg mice, we used the Y maze and Morris water maze. The Tg group showed a decreased percentage of triple alternations compared to the WT group in the Y maze test (*p* < 0.01). The EPOS group had a significantly increased percentage of triple alternations compared with the Tg group (*p* < 0.01), and did not differ from the EGb761 group (Figure 1A), which indicated that EPOS treatment improved spontaneous spatial working memory in 6-month-old 5×FAD Tg mice.

To evaluate spatial learning and memory, we performed the Morris water maze test. During 5 days of training, the mice shortened their time to find the submerged platform, but the escape latency in the Tg group was significantly longer than that in the WT and EPOS groups (Figure 1B, *p* < 0.05). We removed the hidden platform and performed the probe test on the 6th day. Compared with the WT group, the Tg group spent less time in the target quadrant. Treatment with EPOS significantly increased the length of time Tg mice spent in the target quadrant (*p* < 0.05), and time spent in the target quadrant did not differ between the EPOS and EGb761 groups (Figure 1C). These results showed that EPOS ameliorated cognitive deficits in 6-month-old 5×FAD mice.

### Treatment With EPOS Reduced Aβ Plaque Deposition in the Hippocampus

**Figure 2:**
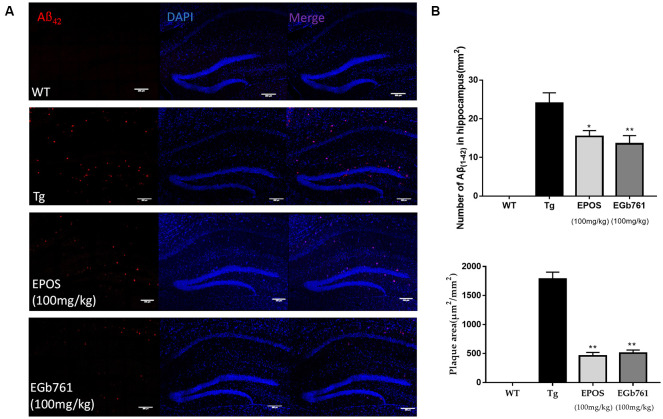
Reduction in Aβ42 plaque deposition in the hippocampus by EPOS. **(A)** Immunofluorescence staining analysis of amyloid-β (Aβ) in the WT, Tg, Tg-EPOS, and Tg-EGb761 groups (magnification 100×). **(B)** Quantitative analysis of the number and volume of Aβ plaques in the hippocampi of the WT, Tg, Tg-EPOS, and Tg-EGb761 groups. *p < 0.05, **p < 0.01 compared with Tg (Mean ± SEM, *n* = 4), Scale bar:100 μm (one-way ANOVA).

To study the effect of EPOS on Aβ accumulation, immunofluorescence staining of Aβ was performed in the hippocampi of 6-month-old mice. The results showed no obvious Aβ plaques in the WT group. In contrast, many plaques were observed in the CA1 of hippocampi in the Tg group (Figure 2A). Compared with the Tg group, the number and area of Aβ plaques were decreased in the EPOS group (*p < 0.05, **p < 0.01; Figure 2B). These results showed that EPOS reduced the size and number of Aβ plaques in the CA1 region of the hippocampus.

### Treatment With EPOS Altered Dendritic Spine Density of Neurons in the Hippocampus

**Figure 3:**
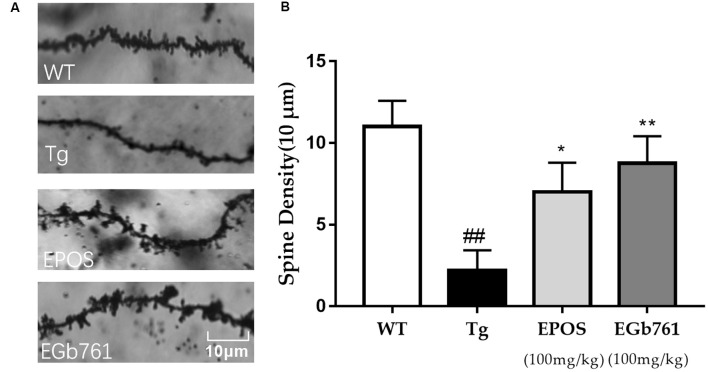
Modulation of dendritic spine density of hippocampal neurons by EPOS. **(A)** Images of Golgi stained dendrites in hippocampi of the WT, Tg, Tg-EPOS, and Tg-EGb761 groups (magnification 100×). **(B)** Bar graphs showing the dendritic spine density. ^##^*P* < 0.01 compared with WT, *p < 0.05 compared with Tg group, **p < 0.01 compared with (Mean ± SEM, *n* ≥ 4), Scale bar: 10 μm (one-way ANOVA).

Dendritic spines are small protrusions on dendrites that play a critical role in synaptic plasticity and cognitive function. Dendritic spine density was calculated from a single nerve in a randomly selected 10-μm region of the hippocampal CA1 region. The morphology of dendritic spines was abnormal in the Tg group (Figure 3A). The densities of dendritic spines were lower in the Tg group compared with those in the WT group. Treatment with EPOS resulted in increased numbers of dendrite spines compared to the 5×FAD Tg group. There were no differences between the EPOS group and the EGb761 group (*p* < 0.05; Figure 3B).

### Fraction 3 of EPOS Most Effectively Delayed Paralysis in the CL4176 *C. elegans* Model of AD

**Figure 4:**
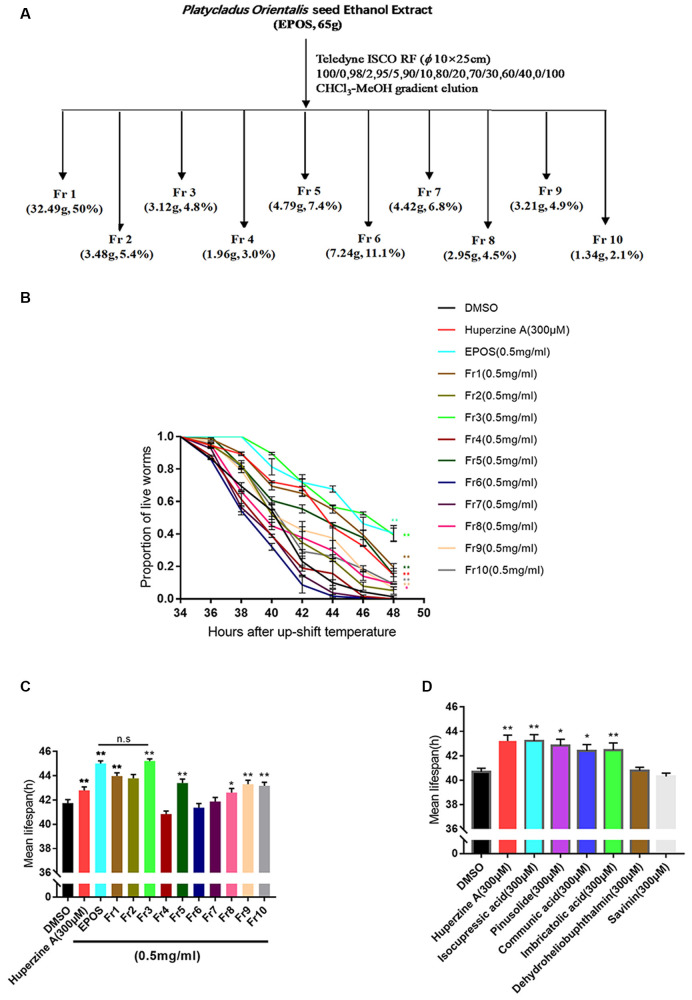
Bioactivity-guided isolation based on delayed paralysis in a CL4176 model of Alzheimer’s Disease (AD). **(A)** Flow chart of fractionation of EPOS using silica gel. **(B,C)** Time course of Aβ-induced paralysis in transgenic *C. elegans* CL4176 treated with standard NGM and each fraction (0.5 mg/ml). Huperzine-A (300 μM) was used as a positive control. **(D)** The mean lifespan of CL4176 treated with standard NGM and compounds. Huperzine-A (300 μM) was used as a positive control. n.s: non significantly compared with EPOS. *p < 0.05 compared with dimethyl sulfoxide (DMSO), **p < 0.01 compared with DMSO (Mean ± SEM, *n* ≥ 60; one-way ANOVA).

Silica gel column chromatography was performed to prepare EPOS fractions (Figure 4A). The activities of the fractions were evaluated using the transgenic *C. elegans* CL4176 strain. As shown in Figures 4B,C, Fr1, Fr2, Fr3, Fr5, Fr8, Fr9, and Fr10 significantly protected worms from Aβ-induced rapid paralysis compared with the untreated control group (*p* < 0.05). The activity of Fr3 did not differ from that of EPOS.

### Compounds in Active Fraction 3 Were Determined Using 1H-NMR (400 MHz), 13C-NMR (100 MHz), and High-Resolution (HR) Electrospray Ionization (ESI)-Mass Spectrometry (MS)

**Figure 5:**
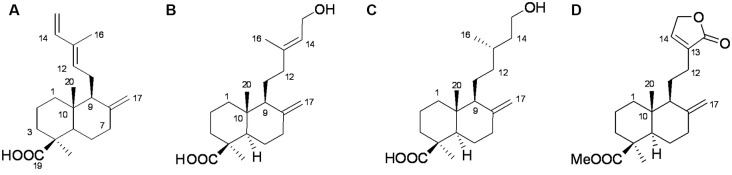
Structures of active compounds. **(A)** Communic acid (HR-ESI-MS m/z 303.2345 [M + H]^+^); **(B)** isocupressic acid (HR-ESI-MS m/z 321.2251 [M + H] +); **(C)** imbricatolic acid (HR-ESI-MS m/z 322.2451 [M + H]^+^); **(D)** pinusolide (HR-ESI-MS m/z 347.2151 [M + H]^+^).

We used ODS column chromatography and preparative HPLC to separate active compounds. We used 1H-NMR (400 MHz), 13C-NMR (100 MHz), and HR-ESI-MS for structural characterization. Communic acid (Kuo and Chen, 1999), isocupressic acid (Fang et al., 1996), imbricatolic acid (Wang et al., 2008), and pinusolide (Kuo and Chen, 1999) were identified (Figure 5, Supplementary Tables S1–S4).

### Active Compounds Isolated From EPOS Attenuated Aβ(1–42) Accumulation in HEK293sw Cells

**Figure 6:**
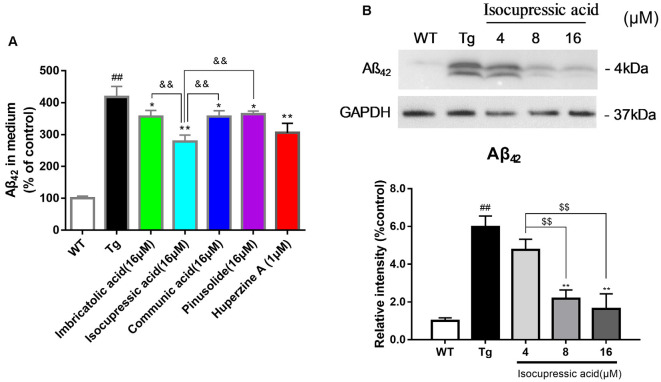
Active components of EPOS attenuated Aβ42 accumulation in HEK293sw cells. **(A)** The effects of treatment with 16 μM communic acid, isocupressic acid, imbricatolic acid, or pinusolide treatment for 24 h on levels of Aβ1–42 in cell culture media were determined using ELISA. **(B)** Levels of Aβ1–42 in cell culture media were determined using western blot. ^##^*p* < 0.01 compared with WT; *p < 0.05, **p < 0.01 compared with Tg; ^$$^*p* < 0.01 compared with 4 μM isocupressic acid group; ^&&^*p* < 0.01 compared with 16 μM isocupressic acid group (Mean ± SEM, *n* = 3; one-way ANOVA).

WT Human Embryonic Kidney 293 cells (HEK293/WT) and HEK293 cells stably transfected with human APP Swedish mutant (HEK293sw) were used to evaluate the effect of isolated active compounds. Treatment with 16 μM communic acid, 16 μM isocupressic acid, 16 μM imbricatolic acid, or 16 μM pinusolide for 24 h resulted in significantly reduced Aβ(1–42)protein levels in HEK293sw cells. Isocupressic acid decreased the levels of Aβ42 to the greatest extent (Figure 6A). Furthermore, Isocupressic acid decreased Aβ(1–42) protein level in a dose-dependent manner (*P* < 0.05; Figure 6B).

### Isocupressic Acid Attenuated Aβ42 Formation *via* BACE1 In HEK293sw Cells

**Figure 7:**
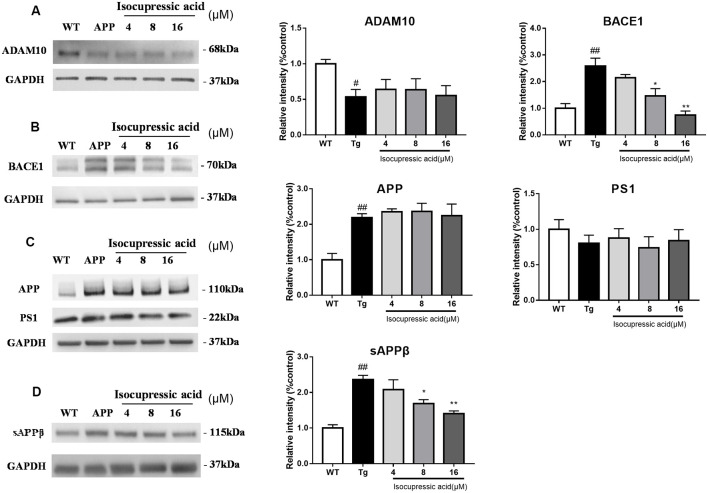
Isocupressic acid attenuated Aβ42 accumulation *via* modulation of BACE1 in HEK293sw cells. Levels of ADAM10 **(A)**, BACE1 **(B)**, APP, and PS1 **(C)**, sAPPβ **(D)** were determined by western-blot analysis. The loading control was glyceraldehyde 3-phosphate dehydrogenase (GAPDH). ^#^*p* < 0.05, ^##^*p* < 0.01 compared with WT. *p < 0.05, **p < 0.01 compared with Tg (one-way ANOVA).

To investigate the effect of isocupressic acid on APP processing, we measured the levels of APP, ADAM10, BACE1, and PS1 using western blot. As shown in Figure 7; the level of BACE1 and sAPPβ protein was higher in HEK293sw cells than in HEK293/WT cells (*P* < 0.01). Furthermore, HEK293sw cells treated with 8 and 16 μM isocupressic acid reduced BACE1 and sAPPβ protein levels (*P* < 0.05). In contrast, treatment with isocupressic acid did not alter the expression of ADAM10, PS1, or APP. These findings indicated that isocupressic acid downregulated Aβ by activating BACE1.

### Isocupressic Acid Reduced BACE1 Activity Through GSK3β/NFκb Pathway in HEK293sw Cells

**Figure 8:**
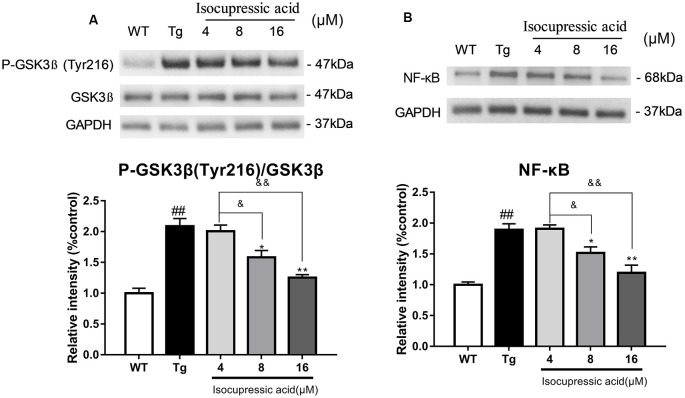
Isocupressic acid reduced BACE1 activity *via* modulation of the GSK3β/NF-κB pathway in HEK293sw cells. **(A)** Phospho-GSK3β, GSK3β, and **(B)** NF-κB protein levels were measured in HEK293-APPsw cell. The loading control was glyceraldehyde 3-phosphate dehydrogenase (GAPDH). ^##^*p* < 0.01 compared with WT. *p < 0.05, **p < 0.01 compared with Tg. ^&^*p* < 0.05, ^&&^*p* < 0.01 compared with 4 μM isocupressic acid (one-way ANOVA).

Western blot was used to evaluate the mechanisms by which isocupressic acid downregulated Aβ and BACE1 in HEK293sw cells. The results showed that 8 μM and 16 μM isocupressic acid significantly inhibited GSK-3β activity and NF-κB expression in HEK293sw cells (*P* < 0.01; Figure 8). These results indicated that isocupressic acid downregulated Aβ and BACE1 in the HEK293sw cells through inhibition of the GSK3β/NF-κB pathway.

## Discussion

Alzheimer’s disease (AD) is the most common age-related progressive neurodegenerative disorder and is characterized by cognitive deficits (Montine et al., 2012). Overproduction of Aβ peptide or reduced clearance is believed to cause AD. The development of strategies to inhibit Aβ aggregation and increase Aβ degradation has received significant attention. Traditional Chinese medicines (TCM), which contain various medicinal components, produce comprehensive effects *via* multiple pathways (Liu et al., 2015). Studies have shown that TCM can be used to prevent and treat neurodegenerative diseases (Wei, 2016). We isolated EPOS and treated 5×FAD mice with this extract to evaluate the anti-AD efficacy of EPOS in comparison to EGb761, which is a well-defined extract of Ginkgo biloba used to treat dementia (von Gunten et al., 2016).

We showed that EPOS significantly improved spatial working memory in 5×FAD mice, as evidenced by a higher spontaneous alternation ratio in the Y maze, and higher quadrant dwell time and shorter escape latency in the Morris water maze (Figure 1). Golgi staining showed that dendritic spine morphology was improved in 5×FAD Tg mice treated with EPOS. The dendritic axes of neurons were thinner and the number of dendritic spines was significantly decreased in 5×FAD Tg mice (Šišková et al., 2014). Treatment with EPOS treatment increased the number of neuronal dendritic spines and improved the morphology of neuronal dendrites, which indicated that EPOS exerted neuroprotection (Figure 3). Studies have shown that Aβ is a neurotoxic protein, and excessive accumulation of Aβ in the brain can lead to neuronal damage, neuronal loss, and destruction of neural networks (Buggia-Prévot et al., 2013). Treatment with EPOS reduced Aβ deposition. Our results showed that EPOS improved cognition and reduced Aβ deposition, which indicated that EPOS may be an effective treatment for AD (Figure 2).

We used modern pharmacodynamic evaluation tools, such as the CL4176 *C. elegans* model and the *in vitro* HEK293sw model, to isolate and identify active compounds. HEK293sw cells were stably transfected with the human APP Swedish mutant (Buggia-Prevot et al., 2008). The transgenic nematode CL4176 strain is a temperature-sensitive mutant strain that expresses human Aβ42 at certain temperatures. Communic acid, isocupressic acid, imbricatolic acid, and pinusolide, which are components isolated from Fr3, have anti-Aβ42 toxicity effect in CL4176 strain (Figure 4D). Besides, these four active compounds inhibit Aβ42 formation in HEK293sw cell (Figure 6A). Furthermore, western blot analysis results showed that isocupressic acid reduced Aβ42 formation *via* decreased expression of amyloidogenic APP pathway (Peineau et al., 2010; Chami and Checler, 2012; Figures 6B, 7).

We then characterized the molecular mechanisms underlying decreased BACE1 expression by isocupressic acid. We investigated whether isocupressic acid influenced BACE1 *via* the GSK-3β/NF-κB pathway. Previous studies showed that NF-κB transcriptionally regulated BACE1 expression (Chen et al., 2012). Also, the activation of GSK3β has been shown to upregulate BACE1 (Ly et al., 2013). Phosphorylation of GSK-3β residues can result in changes in substrate binding affinity. Phosphorylation of Ser9 of GSK-3β results in decrease active site availability, phosphorylation at Tyr216 results in enhanced enzymatic activity. Our results showed that isocupressic acid inhibited GSK3β by decreasing the ratio of pTyr216-GSK3β to total GSK3β, and also reduced NF-κB activity. These results suggested that isocupressic acid suppressed BACE1 expression and Aβ generation *via* the GSK3β/NF-κB pathway (Figure 8).

In summary, for the first time, we found that isocupressic acid is a BACE1 inhibitor, which could inhibit Aβ generation *via* the GSK3β/NF-κB pathway signaling. These findings suggested that *Platycladus orientalis* seed might be a potential source of new anti-AD drugs. However, these conclusions were based on the responses of the HEK293 APPsw cell line and so might not reflect processes in the neuron or neuroblastoma cell lines. In future research, we will study more kinds of neuron cells to further investigate the active compounds in EPSO to improve AD mice cognitive function.

## Data Availability Statement

All datasets generated for this study are included in the article/Supplementary Materials.

## Ethics Statement

All animal experimental procedures in this study were reviewed and approved by the Animal Care and Use Committee of the School of Life Sciences of Sun Yat-sen University (Permission No. 150207).

## Author Contributions

LY conceived and designed the experiments. XH, YJ, JW, FL and RP performed the experiments. XH and YJ analyzed the data. LY and XH prepared the manuscript. PL, YW and WS critically revised this manuscript. All authors contributed to the article and approved the submitted version.

## Conflict of Interest

The authors declare that the research was conducted in the absence of any commercial or financial relationships that could be construed as a potential conflict of interest.
