# Supplementary material for: Modulation of the Aβ-Peptide-Aggregation Pathway by Active Compounds From Platycladus orientalis Seed Extract in Alzheimer’s Disease Models
Source: Front Aging Neurosci. 2020 Aug 14;12:207. doi: 10.3389/fnagi.2020.00207 (PMC7456918; doi:10.3389/fnagi.2020.00207)
Supplement: Supplementary file 1 [file Data_Sheet_1.DOCX]

**Supplementary**

**Table 1** ^1^H-NMR (400 MHz) and^13^C-NMR (100 MHz) data for Communic acid

| Position | communic acid ( in CDCl_3_) | |
| --- | --- | --- |
|  | *δ*_H_ | *δ*_C_ |
| 1 | 1.55, 1.33(2H, m) | 38.7 |
| 2 | 1.52, 1.40(2H, m) | 23.5 |
| 3 | 1.89, 1.56(2H, m) | 38.2 |
| 4 | / | 44.4 |
| 5 | 1.38 (1H, m) | 56.6 |
| 6 | 1.96, 1.75(2H, m) | 26.0 |
| 7 | 2.42, 2.39(2H, m) | 39.5 |
| 8 | / | 148.1 |
| 9 | 1.91 (1H, m) | 56.5 |
| 10 | / | 40.6 |
| 11 | 1.96, 1.86(2H, m) | 29.2 |
| 12 | 5.41(1H, *t, J=*6.0 Hz) | 133.6 |
| 13 | / | 134.1 |
| 14 | 6.32 (1H, *dd, J=*10.8, 17.6 Hz) | 141.8 |
| 15 | 5.04, 4.87 (2H, *d, J=*17.6, 10.8Hz) | 110.1 |
| 16 | 1.75(3H, *s*) | 12.0 |
| 17 | 4.84, 4.47(2H, *s*) | 107.9 |
| 18 | 1.25(3H, *s*) | 20.1 |
| 19 | / | 182.8 |
| 20 | 0.66(3H, *s*) | 13.0 |

**Table 2** ^1^H-NMR (400 MHz) and^13^C-NMR (100 MHz) data for Isocupressic acid

| Position | Isocupressic acid ( in CDCl_3_) | |
| --- | --- | --- |
|  | *δ*_H_ | *δ*_C_ |
| 1 | 1.56, 1.33(2H, m) | 39.2 |
| 2 | 1.50, 1.43(2H, m) | 19.9 |
| 3 | 1.89 (2H, m) | 38.0 |
| 4 | / | 44.2 |
| 5 | 1.33 (1H, m) | 55.6 |
| 6 | 1.96-1.75(2H, m) | 26.1 |
| 7 | 2.41 (2H, m) | 38.4 |
| 8 | / | 147.9 |
| 9 | 1.87 (1H, m) | 56.3 |
| 10 | / | 40.5 |
| 11 | 1.58-1.45 (2H, m) | 22.0 |
| 12 | 1.98(2H, *m*) | 38.7 |
| 13 | / | 140.5 |
| 14 | 5.38 (1H, *t, J=*6.8 Hz) | 123.1 |
| 15 | 4.15(2H, *d, J=*7.2Hz) | 59.4 |
| 16 | 1.67(3H, *s*) | 16.3 |
| 17 | 4.86, 4.53(2H, *s*) | 106.5 |
| 18 | 1.24(3H, *s*) | 28.9 |
| 19 | / | 182.4 |
| 20 | 0.61(3H, *s*) | 12.8 |

**Table 3** ^1^H-NMR (400 MHz) and^13^C-NMR (100 MHz) data for Imbricatolic acid

| Position | Imbricatolic acid ( in CDCl_3_) | |
| --- | --- | --- |
|  | *δ*_H_ | *δ*_C_ |
| 1 | 1.55 (2H, m) | 39.2 |
| 2 | 1.55 (2H, m) | 19.8 |
| 3 | 1.87 (2H, m) | 38.1 |
| 4 | / | 44.1 |
| 5 | 1.31 (1H, m) | 56.4 |
| 6 | 1.96-1.75(2H, m) | 26.1 |
| 7 | 2.42 (2H, m) | 38.8 |
| 8 | / | 148.9 |
| 9 | 1.87 (1H, m) | 56.7 |
| 10 | / | 40.4 |
| 11 | 1.33 (2H, m) | 21.2 |
| 12 | 1.08(2H, *m*) | 36.4 |
| 13 | 1.55 (1H, m) | 30.3 |
| 14 | 1.55 (2H, m) | 39.7 |
| 15 | 3.67(2H, *m*) | 61.3 |
| 16 | 0.90 (3H, *d,J=*6.4Hz) | 19.9 |
| 17 | 4.83, 4.50 (2H, *s*) | 106.4 |
| 18 | 1.24(3H, *s*) | 29.0 |
| 19 | / | 181.6 |
| 20 | 0.60 (3H, *s*) | 12.8 |

**Table 4** ^1^H-NMR (400 MHz) and^13^C-NMR (100 MHz) data for Pinusolide

| Position | Pinusolide ( in CDCl_3_) | |
| --- | --- | --- |
|  | *δ*_H_ | *δ*_C_ |
| 1 | 1.56 (2H, m) | 39.2 |
| 2 | 1.55 (2H, m) | 19.9 |
| 3 | 2.15 (2H, m) | 38.2 |
| 4 | / | 44.3 |
| 5 | 1.59 (1H, m) | 56.3 |
| 6 | 1.97-1.74(2H, m) | 26.2 |
| 7 | 2.42 (2H, m) | 38.6 |
| 8 | / | 147.5 |
| 9 | 1.78 (1H, m) | 55.7 |
| 10 | / | 40.3 |
| 11 | 1.32 (2H, m) | 21.8 |
| 12 | 1.97(2H, *m*) | 24.6 |
| 13 | / | 134.9 |
| 14 | 7.09(1H, t*, J=*1.2Hz) | 143.7 |
| 15 | 4.76(2H, *s*, *J*=2.4Hz) | 70.0 |
| 16 | / | 174.3 |
| 17 | 4.76, 4.58 (2H, *s*) | 106.7 |
| 18 | 1.18(3H, *s*) | 28.8 |
| 19 | / | 177.7 |
| 20 | 0.51 (3H, *s*) | 12.8 |
| 21 | 3.61 (3H, *s*) | 51.1 |
